# A prospective evaluation of ultrasound as a diagnostic tool in acute microcrystalline arthritis

**DOI:** 10.1186/s13075-015-0701-7

**Published:** 2015-07-22

**Authors:** Zufferey Pascal, Roxana Valcov, Isabelle Fabreguet, Alexandre Dumusc, Patrick Omoumi, Alexander So

**Affiliations:** Department of Rheumatology, Département de l’appareil locomoteur, Lausanne University Hospital (CHUV), Av Pierre Decker 5, 1011 Lausanne, Switzerland; Departement de l’appareil locomoteur, Lausanne University Hospital (CHUV), Av Pierre Decker 5, 1011 Lausanne, Switzerland

## Abstract

**Introduction:**

The performance of ultrasound (US) in the diagnosis of acute gouty (MSU) arthritis and calcium pyrophosphate (CPP) arthritis is not yet well defined. Most studies evaluated US as the basis for diagnosing crystal arthritis in already diagnosed cases of gout and few prospective studies have been performed.

**Methods:**

One hundred nine consecutive patients who presented an acute arthritis of suspected microcrystalline arthritis were prospectively included. All underwent an US of the symptomatic joints(s) and of knees, ankles and 1^st^ metatarsopalangeal (MTP) joints by a rheumatologist “blinded” to the clinical history. 92 also had standard X-rays. Crystal identification was the gold standard.

**Results:**

Fifty-one patients had MSU, 28 CPP and 9 had both crystals by microscopic analysis. No crystals were detected in 21. One had septic arthritis. Based on US signs in the symptomatic joint, the sensitivity of US for both gout and CPP was low (60 % for both). In gout, the presence of US signs in the symptomatic joint was highly predictive of the diagnosis (PPV = 92 %). When US diagnosis was based on an examination of multiple joints, the sensitivity for both gout and CPP rose significantly but the specificity and the PPV decreased. In the absence of US signs in all the joints studied, CPP arthritis was unlikely (NPV = 87 %) particularly in patients with no previous crisis (NPV = 94 %). X-ray of the symptomatic joints was confirmed to be not useful in diagnosing gout and was equally sensitive or specific as US in CPP arthritis.

**Conclusions:**

Arthrocenthesis remains the key investigation for the diagnosis of microcrystalline acute arthritis. Although US can help in the diagnostic process, its diagnostic performance is only moderate. US should not be limited to the symptomatic joint. Examination of multiple joints gives a better diagnostic sensitivity but lower specificity.

**Electronic supplementary material:**

The online version of this article (doi:10.1186/s13075-015-0701-7) contains supplementary material, which is available to authorized users.

## Introduction

The gold standard for diagnosing gout (monosodium urate (MSU) arthritis) and calcium pyrophosphate (CPP) arthritis is the identification of crystals in joint fluid [1]. In an acute setting, the sensitivity of crystal identification has been proven to be very high (>90 %), particularly in gout [2, 3]. The performance of this technique is not perfect, however, especially in patients with chronic arthritis and in asymptomatic joints.

Joint aspiration is not always possible, so alternative diagnostic tools have been proposed [4]. These alternative tools include clinical scores [5, 6], ultrasonography [7], magnetic resonance imaging (MRI) [8] and dual-energy computed tomography (DECT) [8, 9]. Ultrasound (US) features of gouty and CPP arthritis have been well described [5, 8, 10, 11], and the technique has been proposed as a convenient diagnostic tool for crystal-induced arthritis [12]. Although the typical US signs of MSU and CPP are well defined [10, 13–17], the accuracy (or reliability) of the technique remains not totally clear. Indeed, the sensitivity (40–95 %) or the specificity (60–95 %) of US for diagnosing both MSU and CPP varied considerably from one study to the other [8, 17, 18]. These differences have several origins: the quality of the equipment, which has greatly improved in the last 5 years; the progressively better recognition of pitfalls, in particular of the double contour sign [19]; the status of the disease (longstanding, acute, chronic) [20, 21]; the type and number of joints examined; the quality of the gold standard (crystal detection in joint aspirate) [3]; and finally the selection of the patients and the controls [22]. Moreover, there have been only a limited number of prospective studies on the performance of US as a diagnostic tool when applied to the setting of acute arthritis [22, 23] in real-life practice. Most US studies have been performed in patients with established disease [22, 24–26].

The primary objective of our study was to assess the performance of US as a diagnostic tool for CPP and MSU in acute crystal arthritis, using crystal identification by microscopy as a gold standard.

The secondary objective was to compare US with X-ray imaging in terms of diagnostic performance.

## Methods

### Selection of patients

We conducted a single-centre prospective study. One hundred and twelve consecutive patients who presented with acute arthritis of suspected microcrystalline origin were included in the study between October 2012 and May 2014. Clinical suspicion of crystal-induced arthritis was based on the following criteria: an acute onset (<10 days duration), swelling of one or few joints (up to three), local and or systemic signs and symptoms of inflammation, and absence of a known diagnosis of inflammatory rheumatic disease such as rheumatoid arthritis, spondyloarthritis or connective tissue disease. Our local ethical committee (Commission d’éthique de la recherche sur l’être humain (CER-VD)) approved the study. Consent to participate to the study was obtained from all patients.

### Clinical data

For each patient, we collected demographic data, history of comorbidities known to be associated with gout (metabolic syndrome, renal impairment, medications that induce hyperuricemia, cardiovascular disease) and CPP (renal insufficiency, calcium/phosphate abnormalities, hypothyroidism). We also recorded previous history of arthritis flares. Because the duration of the illness reflected by previous crisis was suspected to be an important factor influencing the development of US signs, we performed a subset analysis, stratifying the patients into two groups: the former with previous crisis of CCP or MSU arthritis, the latter with none. We also recorded a detailed description of the current clinical presentation: number and localisation of symptomatic joints, duration of symptoms, local and systemic clinical signs such as joint temperature and/or redness presence of extra-articular tophi, C-reactive protein (CRP), uric acid blood levels, ongoing treatments for the current flare, and change in treatment induced by analysis of the synovial fluid.

### Ultrasound

All patients underwent US of the symptomatic joint as well as both knees, ankles and the first metatarsophalangeal (MTP) joints. Each joint was explored using both longitudinal dorsal and lateral approaches. In the knee, a transversal supra-patellar view in maximal flexion was also performed to better examine the condylar cartilage. The machines used were either a Philips HD11 or an Esaote My-lab 70 philips: Best, Netherland Easote. Genoa, Italy. The type of probe was adapted to the size of the joint (from 9 to 18 MHz).

**Fig. 1:**
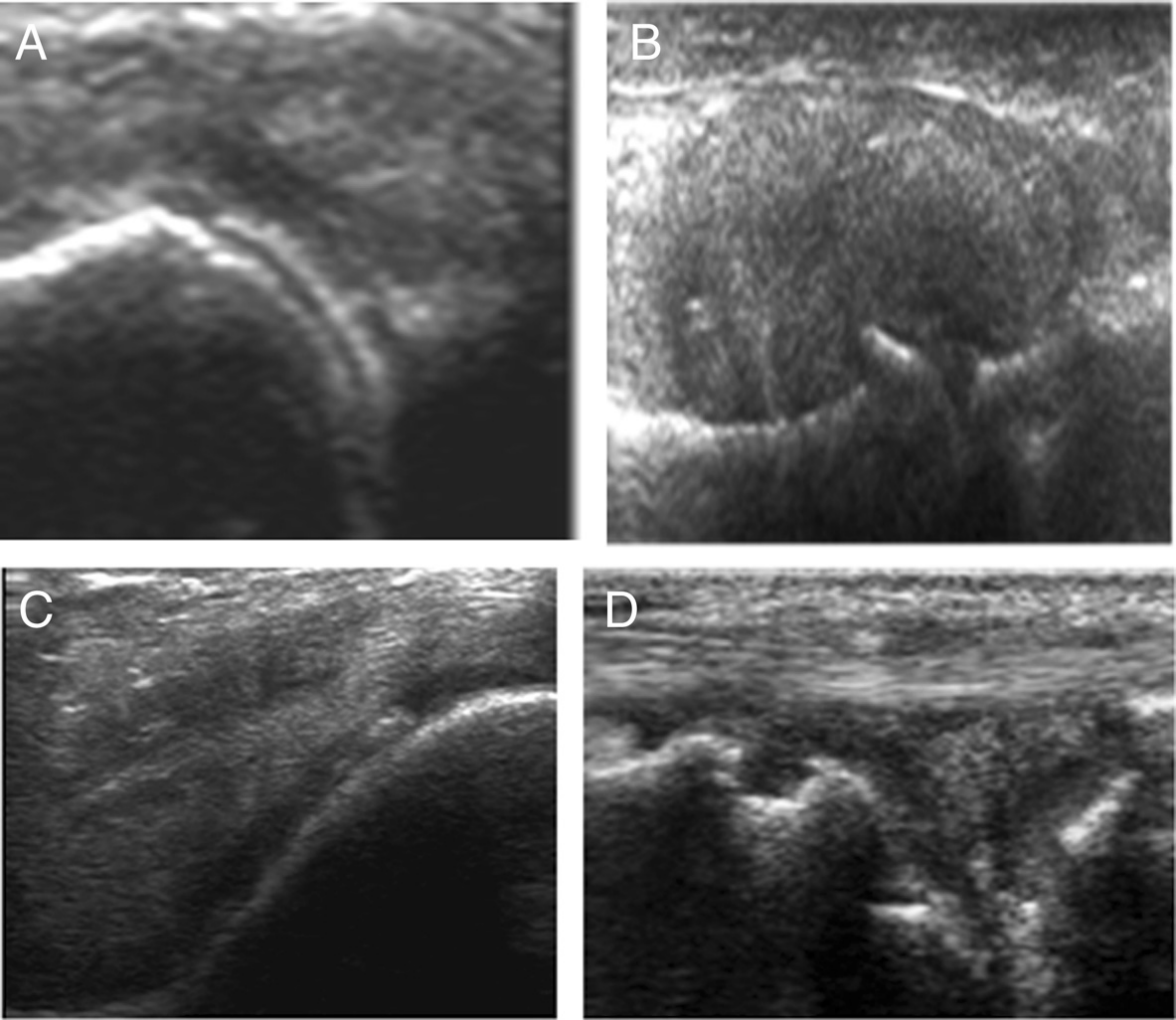
Gout signs: **a** double contour signs and **b** tophus. CPP signs: **c** linear hyperechoic deposit inside the cartilage and **d** in the meniscus

US diagnosis was evaluated based on typical US signs [11, 24]. For gout, we took into account the ‘double contour sign’ (Fig. 1a) and/or the presence of tophi, defined as a non-homogeneous mass that could be hypoechoic or hyperechoic, surrounded by a small anechoic rim (Fig. 1b). Hyperechogenic spots considered as aggregates of crystals were not considered. For CPP, intra-cartilage (Fig. 1c), meniscus (Fig. 1d), synovial or tendon hyperechoic deposits were considered diagnostic. Other US signs not necessarily relevant for the diagnosis were also recorded. They included erosions, osteophytes and Doppler activity in the synovium or around tophi. Symptomatic joints were examined first, followed by all other joints.

Two rheumatologists who were not aware of the clinical history or examination performed all US examinations. One rheumatologist has over 15 years of experience, and the other 2 years of experience. To determine the inter-observer agreement, the first 10 patients were examined in common by the two operators.

### Joints aspiration and examination

Joint aspiration for microbiology and crystal analysis was performed on the symptomatic joints. Many of the aspirations were carried out under US guidance, especially when small joints were symptomatic or when little synovial fluid was present. US and joint aspiration were performed usually at the same time, but at most within 24 hours of each other.

Identification of crystals was based on crystal birefringence and crystal morphology. Only intracellular crystals were taken into account for diagnosis. The diagnosis was confirmed by two different observers (one directly after the puncture by the clinician; the other a technician in the laboratory dedicated to joint fluid analysis). In the laboratory, the fluid was first centrifuged in order to enhance the concentration of cells and of potential crystals. In the 10 cases where the amount of liquid was too small to be sent to the laboratory, the diagnosis was only based on clinician observation. In three cases of disagreement between the two observers, the laboratory diagnosis was retained.

A synovial fluid culture was also performed in each case to exclude septic arthritis. When the amount of fluid was adequate, a numeration of leukocytes was also performed to confirm the inflammatory nature of the acute flare.

### X-ray imaging

X-ray imaging were recommended but not mandatory. Available X-ray imaging at the time of the acute attack were compared with US findings. Symptomatic and non-symptomatic joints were analysed separately. X-ray imaging were reviewed in consensus by two different rheumatologists as well as a musculoskeletal radiologist, all blinded to the final diagnosis. Radiological signs of gout were well-defined juxta-articular sclerotic erosions ± localised soft tissue swelling compatible with tophi, and CPP signs were linear radiopaque deposits projecting on articular soft tissues, in particular the cartilage, meniscus, tendons or synovium.

### Statistical analysis

Results for quantitative clinical and demographic variables are reported as the mean ± standard deviation, and results for qualitative variables as numbers or percentages per category. The groups were compared by univariate logistic regression. *P* <0.05 was considered significant.

US and X-ray diagnoses were considered as index tests, with crystal identification in the fluid analysis considered the referent test for the diagnosis. The performance of US and X-ray imaging was evaluated by assessing the sensitivity, specificity, positive predictive value (PPV) and negative predictive value (NPV) on symptomatic joints and also in all joints. Tests were performed using statistical software (MedCalc Software, Ostend, Belgium).

## Results

### Clinical and demographic data

One hundred and twelve patients fulfilled the inclusion criteria; 109 of these were eventually analysed after the exclusion of three patients due to the absence of fluid on puncture. The demographic data for the 109 remaining patients are summarised in Table 1. Sixty-two patients had monoarthritis and 47 had oligoarthritis. Monoarthritis was more frequent in patients with CPP arthritis (Table 1). Patients were predominately older males in all groups. In 40 % of MSU and in 70 % of CPP diagnosed arthritis cases, the presenting attack was the first reported symptomatic episode of crystal arthritis. There were a high number of comorbidities but not of the same type in gout and non-gout patients (more cardiovascular diseases, hyperlipidaemia, diabetes in gout patients against more renal insufficiency, hypothyroidism in CPP patients). The mean CRP was elevated in all groups but was significantly higher in gout patients (Table 1). This was also the case for serum uric acid levels measured at the time of the attack.

**Table 1:** Demographic and clinical data for the patients according to the diagnosis based on the analysis of joint fluid

	MSU (*n* = 60)^a^	CPP (*n* = 37)^a^	No crystals (*n* = 21)	*P* value
Sex (% male)	92	68	79	NS
Age (years), mean (SD)	65 (12)	75 (15)	67 (10)	NS
First attack of arthritis (%)	41	70	67	NS
Monoarticular arthritis (*n*)	31	26	11	<0.05^b,c^
Oligoarticular arthritis (*n*)	29	11	10	<0.05*
CRP (mg/l), mean, *n* = 10 mg/l (SD)	108 (77)	76 (84)	46 (42)	<0.05^d^
Urate (μmol/l), mean (SD)	477 (160)	335 (113)	390 (131)	<0.05^e^

*CPP* calcium pyrophosphate, *CRP* C-reactive protein, *MSU* monosodium urate, *NS* not significant, *SD* standard deviation

^a^It was hard to determine which crystal was responsible for the acute flare, so the nine patients with both crystals were added to both the MSU (gout) and CPP (chondrocalcinosis) groups

^b^No crystal versus CPP and MSU

^c^CCP versus gout and no crystal

^d^No crystal versus gout

^e^CCP versus gout

### Puncture and fluid analysis

**Fig. 2:**
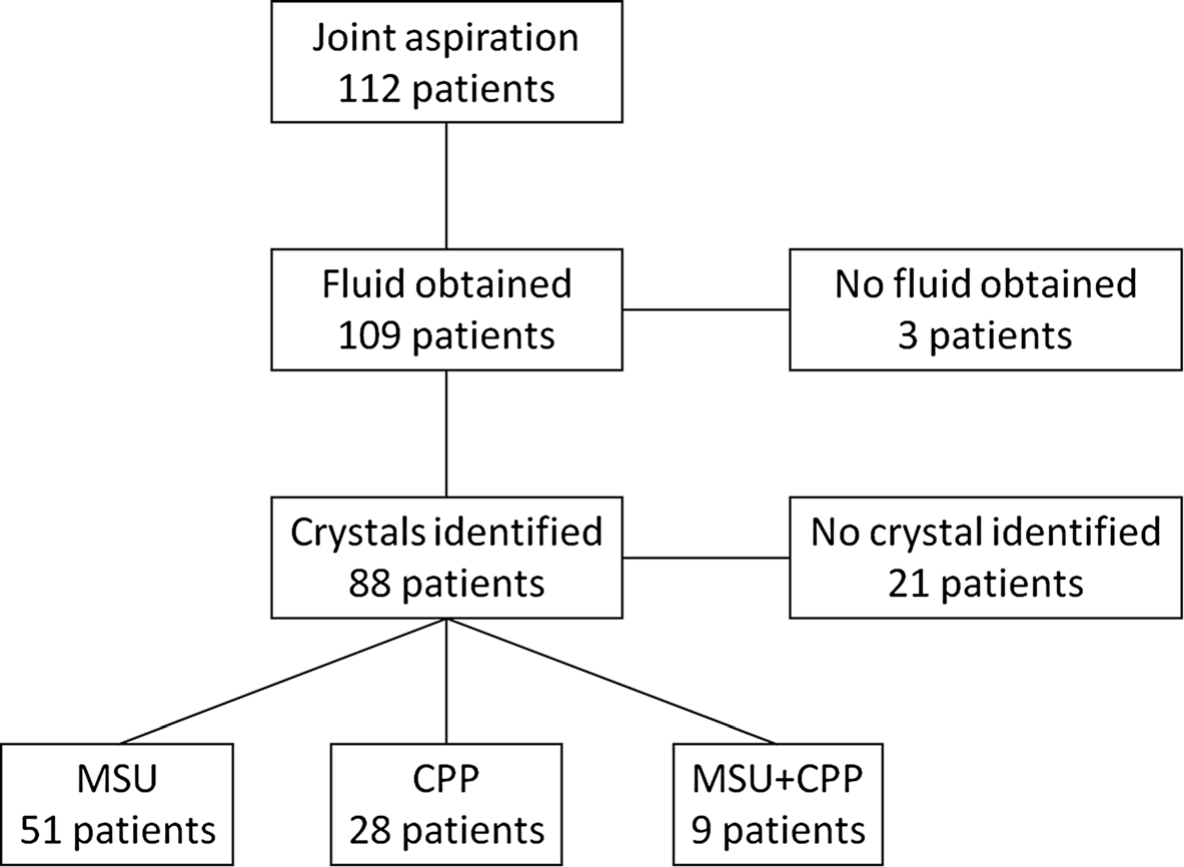
Distribution of the patients according to crystal fluid analysis. *CPP* calcium pyrophosphate, *MSU* monosodium urate

Results of crystal analysis in the fluid aspirations are decrypted in Fig. 2. MSU crystals were found in 51 patients and CPP crystals in 28 patients. Twenty-one patients had no crystals. Both MSU and CPP crystals were detected in the same symptomatic joint in nine patients. In these subjects it was impossible to determine which crystal was responsible for the acute flare, so we added them to both groups (total of 118: MSU, *n* = 60; CPP, *n* = 37; no crystal, *n* = 21).

The knee was the most common symptomatic joint, followed by the metatarsal, the ankle joint and the wrist. Crystals were also most frequently identified thanks to fluid aspiration in the knees for both MSU arthritis (27/60 positive cases) and CPP arthritis (22/37 positive cases). The latter crystal was never found in the first MTP joints, contrary to MSU crystals. A movie file shows this in more detail (see Additional file 1).

The design of the study did not permit collection of supplementary investigations and follow-up in the 21 patients without crystals. At the time of joint aspiration, the diagnosis of a non-specific inflammatory arthritis was retained in 12 of these patients, based on the synovial white cell count. Microbiology was negative except for one patient who was treated for septic arthritis. In this patient, joint fluid aspiration did not reveal any crystals and no US signs of MSU or CPP arthritis were detected. Three patients had a non-inflammatory fluid collection.

### Ultrasound

US signs of microcrystalline arthritis (MSU and/or CPP) were present in 65/88 patients with crystals detected in the joint aspirations (sensitivity of 74 %), and only in 3/21 patients with no crystals identified (specificity of 82 %).

The utility of US signs for the diagnosis of MSU and CPP arthritis was evaluated separately: firstly by considering the presence of US signs for each form of arthritis in the presenting symptomatic joint only; and secondly by the presence of US signs in all joints examined.

When the analysis was limited to US signs in the target joints, the sensitivity of US for gout was 60 % (44 % for ‘double contour’ only) and also 60 % for CPP arthritis. When US signs of other joints were taken into account, the US sensitivity for MSU rose from 60 to 84 % and for CPP from 60 to 81 %. However, the specificity went down from 92 to 78 % for gout and from 80 to 62 % for CPP (Table 2).

**Table 2:** Sensitivity, specificity, positive predictive value and negative predictive value of ultrasound signs for MSU and CPP arthritis

	MSU symptomatic joint	MSU multiple joints	CPP symptomatic joint	CPP multiple joints
Sensitivity (%)	60	84	60	81
Specificity (%)	92	78	80	62
Positive predictive value (%)	92	82	60	52
Negative predictive value (%)	62	77	80	87

The nine patients with both crystals were added to both the MSU and CPP groups

*CPP* calcium pyrophosphate, *MSU* monosodium urate

In the presence of US MSU deposition signs in the symptomatic joint, gouty arthritis was highly probable (PPV >90 %). This was not the case for CPP (PPV = 60 %).

In the absence of any US signs of CPP after multiple joints examination, CPP arthritis was unlikely (NPV = 87 %). This was less in the case of gout (NPV = 77 %) (Table 2).

The sensitivity and the PPV of US limited to the symptomatic joints for both MSU and CCP were not significantly influenced by a history of previous crisis. A file shows this in more detail (see Additional file 2). The performances of multiple joint examinations were enhanced (see Additional file 2). However, only in patients with no previous history of CCP arthritis could the absence of US signs rule out CCP diagnosis (NPV = 94 % against 50 % in absence of previous crisis).

The first MTP joint was the most common site of US signs for gout in symptomatic and asymptomatic joints (Table 3). For CPP, most of the signs were present essentially in the knees and in the wrists. US CPP signs of crystal deposition especially in the knee were present in many patients with no CPP crystal-proven arthritis (Table 3), explaining the low specificity of US in chondrocalcinosis.

**Table 3:** Frequency of ultrasound signs in patients according to the joints examined

	Joint localisation of ultrasound signs (number/joint)^a^					
	Knee	MTP	Wrist	Ankle	Hand	Others
Gout confirmed (60 patients)						
Symptomatic joints						
MSU signs	10	38	0	9	1	0
CPP signs	13	2	0	1	1	0
Asymptomatic joints^b^						
MSU signs	10	32		9		
CPP signs	12	5		3		
CPP confirmed (37 patients)						
Symptomatic joints						
CPP signs	22	1	4	1	1	0
MSU signs	0	5		3		
Asymptomatic joints						
CPP signs	15	7		1		
MSU signs	0	7		3		
No crystals (21 patients)						
Symptomatic joints						
MSU sign	0	2	0	0	0	0
CPP sign	3	1	2	0	0	0
Asymptomatic joints						
MSU signs	0	3		0		0
CPP signs	5	3		1		0

*CPP* calcium pyrophosphate, *MSU* monosodium urate, *MTP* metatarsophalangeal joint

^a^For some patients several joints were symptomatic, so the number of joints is larger than the number of patients

^b^For some patients signs were present on symptomatic and asymptomatic joints, so the number of joints is larger than the number of patients

In the subgroup of patients (*n* = 9) with both crystals, US was of little help in the differential diagnosis between CPP and MSU arthritis. US signs of both diseases were present in symptomatic joints of three patients, MSU signs only in two patients and CPP signs in four patients. When US was performed on multiple joints, all patients had US signs of at least one or both diseases.

### X-ray imaging

X-ray imaging of the affected joint were not mandatory for this study. Eighty of 109 patients had an X-ray scan of the symptomatic joint and 92/109 patients had X-ray imaging for at least one of the other asymptomatic joints evaluated by US. In gout patients (40 patients), X-ray imaging of the symptomatic joints was confirmed not to be useful because of the very poor sensitivity and NPV (see Table 4).

**Table 4:** Sensitivity, specificity, positive predictive value and negative predictive value of X-ray imaging for MSU and CPP arthritis

	MSU symptomatic joint	MSU multiple joints	CPP symptomatic joint	CPP multiple joints
Sensitivity (%)	5.6	19	55	58
Specificity (%)	100	100	82	79
Positive predictive value (%)	100	100	65	64
Negative predictive value (%)	53.8	55	77	78

The nine patients with both crystals were added to both the MSU and CPP groups

*CPP* calcium pyrophosphate, *MSU* monosodium urate

In CPP arthritis, the performance of X-ray and US imaging on the symptomatic joints in the 29 patients for whom both procedures were available were similar: sensitivity of 55 % versus 63 %, and specificity of 82 % versus 90 % respectively. As the US results matched those of the overall cohort (Table 2), the performance of X-ray imaging can be to could be extrapolate to the entire cohort.

Adding X-ray imaging of additional joints did not change significantly the sensitivity or the specificity for both CPP and MSU arthritis (Table 4). Because the number and the localisation of additional joints examined by radiography were not always similar, it was impossible to strictly compare the performance of X-ray imaging and US.

In the subgroup of patients with double crystals found in the joint fluid, 4/6 patients had radiographic signs of chondrocalcinosis.

## Discussion

The primary objective of our study was to determine whether the diagnostic performance of US was sufficiently good to allow the clinician to skip joint aspiration and crystal identification by microscopy in order to make a diagnosis of microcrystal-induced arthritis. This situation is frequently encountered in clinical practice in the primary or secondary care setting and we designed our study to fit as closely as possible these real-life conditions.

Our results showed that US signs of either MSU or CPP alone are not sensitive enough as a diagnostic tool. When US was limited to the symptomatic joints, its sensitivity was only around 60 %. However, when US signs of gout were present they were highly predictive of the diagnosis of MSU arthritis (PPV = 92 %). This was much less the case for acute CPP arthritis. Indeed, on the contrary to MSU arthritis, the positive predictive value of US CPP signs in the symptomatic joints was low (PPV = 60 %). This could be partially explained by the high prevalence of chondrocalcinosis in our patients, who were by and large an older population.

By examining non-symptomatic joints as part of the US examination, the sensitivity of the test was enhanced but the specificity decreased. In CPP arthritis, when no US signs were found after examination of all the pre-specified joints, the disease could be reasonably ruled out (NPV = 87 %). The same result has been confirmed by other groups using the same approach, suggesting that US examination should not be limited to symptomatic joints only [25]. However, there is still no consensus on which additional joints should be scanned. According to our study and the literature, examination of the knee is mandatory for CPP arthritis [20] and the MTP joint for MSU. Some studies suggest that the wrist should also be examined [16].

The justification for requesting an X-ray scan of the symptomatic joint when one suspects an acute microcrystalline arthritis is debatable. Our study confirmed that X-ray imaging has no place as a diagnostic tool for MSU arthritis [27, 28] and does not perform better than US for CPP [11]. In this latter situation, we think that US should be the first—and in most settings the unique—imaging modality, as suggested by other authors [11].

Our results need some additional comments with regard to their interpretation and the study has some limitations. Owing to our study design, no definite diagnosis of non-crystal arthritis could be assigned in 20 % of the cases. We cannot rule out that some of these patients in this group indeed had a microcrystalline arthritis (either MSU or CCP arthritis) that could have been confirmed if followed up, as demonstrated in some studies [14].

The diagnostic performance of US for microcrystalline arthritis appears to be lower in our study than in some recent publications [16]. This difference can partly be explained by the duration of the illness. Indeed, in many of our patients the acute arthritis was the first flare and it is known that typical US signs accumulate in the course of the disease for either MSU or CPP disease. This was only partially confirmed when we stratified our patients according to the presence or not of previous crisis. For MSU, we were also strict in the interpretation of a positive ‘double contour’ sign. This sign was retained only when the hyperechoic deposit was irregular and present not only directly perpendicular under the probe but independent from the insonation angle in order to differentiate from reinforcement artefacts. Moreover, hyperechogenic spots—considered by some authors as aggregates of MSU crystals—were not taken into account because this sign is not yet confirmed to be specific for MSU arthritis [29, 30].

Although two different clinicians looked for the presence of crystals, we cannot exclude some cases of detection errors, which would partially explain the low sensitivity, especially for CPP (see Table 4). Previously, a high sensitivity of US for CPP had been demonstrated essentially for the knee joint [10], but in our study almost one-half of the symptomatic CPP flares concerned other joints. We also had a low number of controls without crystals compared with other studies due to the profile of the study including patients with a high suspicion of microcrystalline disease. This selection and the quite elevated mean age of the patients certainly explained why we found both crystals in nine of them, essentially in the knee where the prevalence of symptomatic and/or asymptomatic chondrocalcinosis is high. Finally, the advances in US technology will certainly improve to demonstration of crystal deposits and therefore enhance the sensitivity of US for microcrystalline arthritis, thanks to higher frequency probes and optimisation of the software.

The treatment for an acute flare of CPP [31] and MSU [32] arthritis is in most cases very similar, and therefore the absolute necessity of a precise diagnosis for initiating an immediate treatment strategy is not critical [3, 7]. This remark applies also to the patients with signs of both conditions at US and even on synovial fluid analysis. In our study, however, we found one case of septic arthritis with no US signs of microcrystalline arthropathy. In the very acute phase, there are unfortunately no clinical, US or radiological elements can that can clearly differentiate septic arthritis from microcrystalline arthritis [33]. Joint aspiration therefore remains, for us, absolutely necessary when septic arthritis is suspected. If the clinician decides to start, based on US data, a treatment for crystal arthritis without a joint puncture, the patient should be carefully followed up and this attitude should be rapidly revised in the case of a non-response.

## Conclusions

Our study confirms that arthrocentesis remains the key investigation for the diagnosis of microcrystalline acute arthritis. The exact place of US in the diagnosis of acute microcrystalline arthritis is still not well defined [30]. The results of our study suggest that US is much more useful than X-ray imaging. US can be a real help for puncture in small joints and could serve as an alternative procedure, in case of impossibility or contraindication to joint aspiration. The presence of MSU signs on the symptomatic joint is highly suggestive for the diagnosis of gout. US should not, however, be limited to the symptomatic joint. US of multiple joints give a better diagnostic sensitivity but lower specificity. In the absence of US signs of CPP on multiple joint US, it is highly unlikely that arthritis is due to CPP in patients with no previous history of such disease.

## Additional files

Additional file 1:
**Number of joints: symptomatic, punctured and according to the diagnosis after arthrocentesis.**
Additional file 2:
**Sensitivity, specificity, positive predictive value (PPV), negative predictive values (NPV) of X-rays for MSU and CPP arthritis patients with previous crisis.**

## Abbreviations

CER-VD: Commission d’éthique de la recherche sur l’être humain
CPP: Calcium pyrophosphate
CRP: C-reactive protein
DECT: dual-energy computed tomography
MRI: Magnetic resonance imaging
MSU: Monosodium urate
MTP: Metatarsophalangeal
NPV: Negative predictive value
PPV: Positive predictive value
US: Ultrasound
