# Erratum to: A prospective evaluation of ultrasound as a diagnostic tool in acute microcrystalline arthritis

**DOI:** 10.1186/s13075-015-0746-7

**Published:** 2015-09-16

**Authors:** Pascal Zufferey, Roxana Valcov, Isabelle Fabreguet, Alexandre Dumusc, Patrick Omoumi, Alexander So

**Affiliations:** Department of Rheumatology, Département de l’appareil locomoteur, Lausanne University Hospital (CHUV), Av Pierre Decker 5, Lausanne, 1011 Switzerland; Departement de l’appareil locomoteur, Lausanne University Hospital (CHUV), Av Pierre Decker 5, Lausanne, 1011 Switzerland

Unfortunately, the original version of this article [1] contained an error. The first author name was included incorrectly. The correct first author name is Pascal Zufferey. This has been corrected in the original article and the correct author list is also included correctly below.

Pascal Zufferey^1*^, Roxana Valcov^1^, Isabelle Fabreguet^1^, Alexandre Dumusc^1^, Patrick Omoumi^2^ and Alexander So^1^
