# Supplementary material for: A prospective evaluation of ultrasound as a diagnostic tool in acute microcrystalline arthritis
Source: Arthritis Res Ther. 2015 Jul 22;17(1):188. doi: 10.1186/s13075-015-0701-7 (PMC4511437; doi:10.1186/s13075-015-0701-7)
Supplement: Additional file 1: — Number of joints: symptomatic, punctured and according to the diagnosis after arthrocentesis. [file 13075_2015_701_MOESM1_ESM.docx]

Additional file1 :

| **joints** | **Symptomatic*** | **punctured** | **CCP** | **MSU** |
| --- | --- | --- | --- | --- |
| knee | 34 | 55 | 22 | 27 |
| Metatarso phallangeal | 27 | 16 | 0 | 10 |
| Tibio tarsal | 19 | 20 | 3 | 15 |
| wrist | 14 | 16 | 8 | 3 |
| Others | 7 | 0 | 4 | 1 |
| hand | 5 | 5 | 0 | 5 |

**Number of joints: symptomatic, punctured and according to the diagnosis after arthrocentesis**
