# Supplementary material for: A prospective evaluation of ultrasound as a diagnostic tool in acute microcrystalline arthritis
Source: Arthritis Res Ther. 2015 Jul 22;17(1):188. doi: 10.1186/s13075-015-0701-7 (PMC4511437; doi:10.1186/s13075-015-0701-7)
Supplement: Additional file 2: — Sensitivity, specificity, positive predictive value (PPV), negative predictive values (NPV) of X-rays for MSU and CPP arthritis patients with previous crisis. [file 13075_2015_701_MOESM2_ESM.docx]

Additional file2

Sensitivity, specificity, positive predictive value (PPV), negative predictive values (NPV) of X-rays for MSU and CPP arthritis patients with no previous crisis

|  | MSU  Symptomatic  Joint | MSU  Multiple  Joints | CPP  Symptomatic  Joint | CPP  Multiple  Joints |
| --- | --- | --- | --- | --- |
| Sensitivity : % | 61 | 69 | 71 | 88 |
| Specificity % | 94 | 91 | 85 | 70 |
| PPV% | 87 | 84 | 60 | 53 |
| NPV% | 78 | 82 | 91 | 94 |

Sensitivity, specificity, positive predictive value (PPV), negative predictive values (NPV) of X-rays for MSU and CPP arthritis patients with previous crisis :

|  | MSU  Symptomatic  Joint | MSU  Multiple  Joints | CPP  Symptomatic  Joint | CPP  Multiple  Joints |
| --- | --- | --- | --- | --- |
| Sensitivity : % | 56 | 89 | 63 | 81 |
| Specificity % | 94 | 89 | 40 | 40 |
| PPV% | 95 | 94 | 68 | 75 |
| NPV% | 47 | 80 | 33 | 50 |
